# The impact of pectoralis major fascia preservation on postoperative quality of life and shoulder function in endoscopic thyroidectomy via axillary approach

**DOI:** 10.3389/fendo.2025.1669340

**Published:** 2025-11-14

**Authors:** Hang Xu, Sihan Li, Xulin Wang, Benlong Zhang, Yaqi Wang, Chang Lu, Cheng Deng, Shangtong Lei

**Affiliations:** 1 Department of Breast Surgery, Haikou People’s Hospital, Haikou Affiliated Hospital of Central South University Xiangya School of Medicine, Haikou, China; 2 Department of General Surgery, Nanfang Hospital, Southern Medical University, Guangzhou, China

**Keywords:** endoscopic thyroidectomy, pectoralis major fascia, postoperative recovery, shoulder function, THYCA-QoL, ASES score

## Abstract

**Background:**

The impact of pectoralis major fascia (PMF) preservation during endoscopic thyroidectomy (ET) via axillary approach on postoperative recovery remains poorly understood. This study aimed to compare the quality of life (QoL) and shoulder function between patients with and without PMF preservation intraoperatively.

**Methods:**

A total of 77 patients were enrolled, including 39 cases with the PMF preservation (Group A) and 38 cases without (Group B). Postoperatively QoL and shoulder joint function were assessed at 1month, 3 months, and 6 months using Thyroid Cancer-Specific Quality of Life (THYCA-QoL) questionnaire and American Shoulder and Elbow Surgeons Standardized Shoulder Assessment Form (ASES) questionnaire, respectively.

**Results:**

The median follow-up time was 7.55 ± 1.36 months across all cases. Intraoperatively, Group A exhibited significantly lower total drainage volume than Group B (p <0.001). During postoperative follow-up, while THYCA-QoL scores were comparable at 1 and 6 months, Group A demonstrated superior neuromuscular (p = 0.03), sympathetic (p = 0.01), and sensory (p = 0.01) recovery at 6 months. ASES scores revealed no differences at 1 month, however, by 3 months, Group A achieved higher total scores (p = 0.02). At 6 months, Group A outperformed Group B in total ASES score (p < 0.001), pain (p = 0.04), and function (p < 0.001).

**Conclusion:**

Preserving the PMF during ET via an axillary approach can improve QoL, reduce bleeding, enhance long-term sensory and shoulder functional recovery, suggesting that the protection of PMF might have a positive impact on the postoperative patient recovery.

## Introduction

Endoscopic thyroidectomy (ET) has emerged as a minimally invasive alternative to conventional open thyroidectomy, offering superior cosmetic outcomes, reduced postoperative pain, and faster recovery (1, 2). Among various endoscopic approaches, ET via axillary approach stands out due to its excellent cosmetic results, as the incision is concealed within the axillary skin folds, leaving no visible neck scars (2). However, the transaxillary approach requires dissection through the pectoralis major fascia (PMF) to access the thyroid region, which may result in fascial injury and potentially impair shoulder joint movement. Therefore, preservation of the PMF during the ET via axillary approach is crucial for the maintaining the quality of life (QoL) of patients. Nevertheless, no studies have systematically evaluated the impact of PMF preservation on the QoL and shoulder joint function in these patients.

Thus, the purpose of this study was to evaluate the impact of PMF preservation on QoL and shoulder joint function in patients undergoing ET via an axillary approach.

## Methods

### Patients

In this retrospective cohort study, patients undergoing ET via axillary approach for benign or malignant thyroid masses were recruited from Department of Breast Surgery, Haikou People’s Hospital between February 2024 to June 2025. All operations were conducted by the same surgical team. The indications for surgery in this cohort were standardized as follows: Malignant nodules: cytologically or histologically confirmed papillary thyroid carcinoma (PTC), or nodules meeting ATA high-suspicion sonographic criteria for PTC when patients refused fine-needle aspiration biopsy, limited to T1N0M0 stage disease. Benign nodules: nodules < 4 cm in maximum diameter associated with compressive symptoms such as dysphagia, dyspnea, or significant local discomfort.

**Figure 1:**
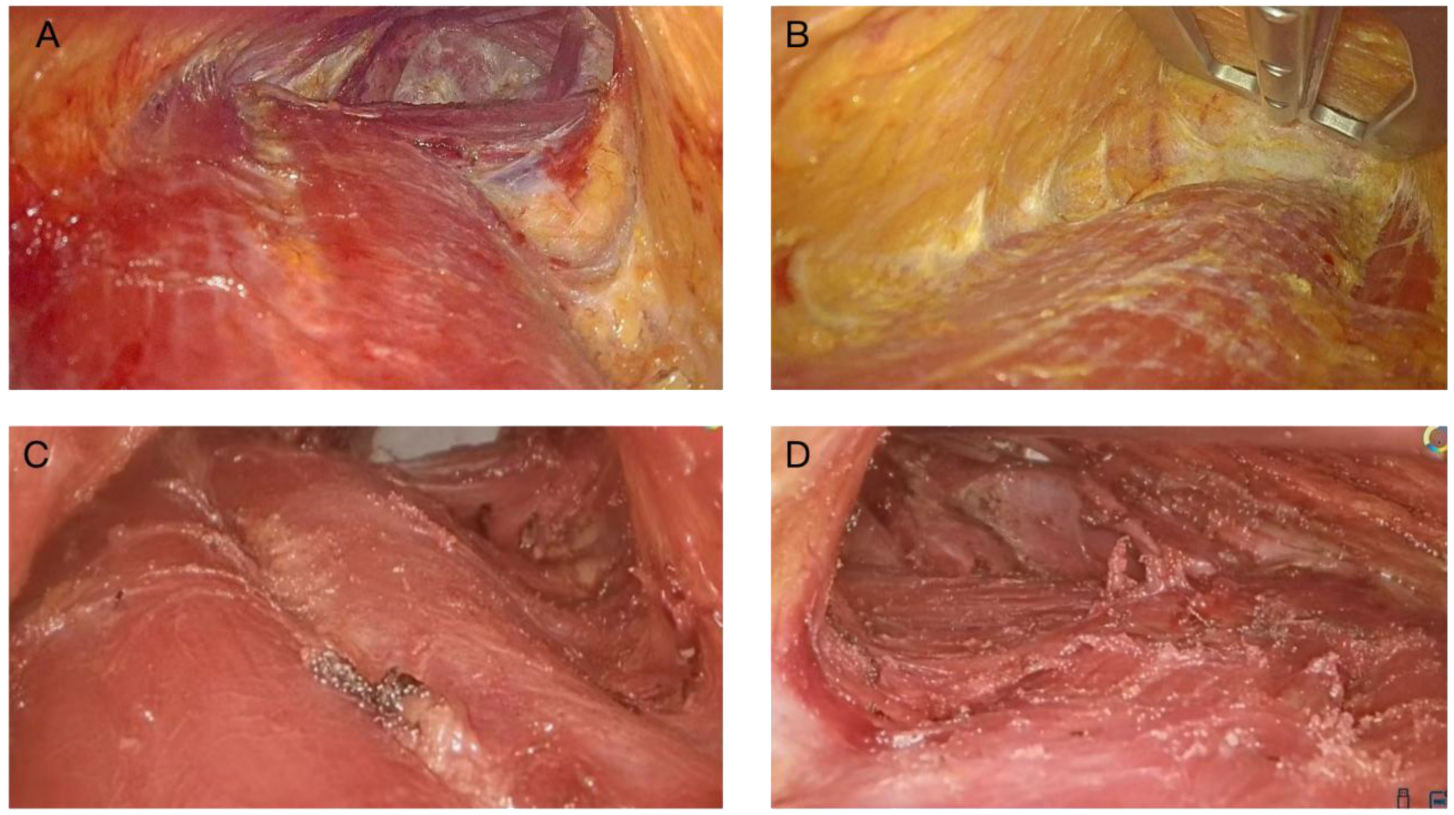
Intraoperative assessment of pectoralis major fascia (PMF) integrity: preserved vs. non-preserved. Representative intraoperative images demonstrating **(A, B)** an intact PMF used as the dissection/gliding plane **(C, D)** a disrupted PMF with exposure of underlying muscle fibers.

Using the surgical video analysis, we categorized patients into two groups based on PMF preservation status: the PMF-preserved group (Group A) and the non-PMF-preserved group (Group B) (**Figure 1**). Two independent surgeons (XH and LS) were evaluated videos and scored based on the extent of PMF preservation. Preserved PMF was defined as continuous fascia covering the pectoralis major muscle, including cases with only small focal defects (<2 cm²). Non-preserved PMF was defined as clear fascial discontinuity with broad exposure of underlying muscle fibers (≥2 cm²) (3–5).

Exclusion criteria included thyroiditis or hyperthyroidism, history of neck/chest surgery or trauma, diabetes, mental disorders, refusal to provide information consent, incomplete 6-month follow-up or questionnaire data, failed supraclavicular nerve exposure, conversion to open surgery, postoperative hematoma, and requirement for lateral neck dissection. Patients with diabetes were excluded because it is associated with impaired wound healing and higher surgical-site infection risk, and may independently worsen neurosensory symptoms and shoulder function (6–8). All patients provided written informed consent for participation and the study was approved by Institutional Review Board.

### Operative technique

The operative procedure was same as previously described (9). A 4-cm incision was made along the right axillary skin line, and the skin and subcutaneous tissue were incised. The light source and auxiliary retractor were positioned on the surface of the PMF, ensuring minimal disruption and preservation of its integrity. A flap was carefully elevated up to the level of the thyroid cartilage, maintaining the underlying fascial attachments as much as possible. Under endoscopic guidance, access to the sternocleidomastoid muscle space was established, followed by dissociation of the scapulohyoid muscle and anterior cervical muscle group to reach the anterior aspect of the trachea. Then, with utmost care, one lobe of the thyroid gland was turned inward while ensuring protection of the recurrent laryngeal nerve throughout this process. Finally, the unilateral thyroid gland lobe along with its isthmus was completely excised. Lymph node dissection was continued in both the central compartment and the lateral areas, encompassing the common carotid artery on one side and the front of the trachea on the other, extending to the tracheoesophageal groove at the back; lymph nodes situated behind the recurrent laryngeal nerve were also meticulously dissected, along with any lymph nodes or adipose tissue above the thymus tissue (**Figure 1**).

### Questionnaire

A quality of life (QoL) questionnaire was assessed using the Thyroid Cancer-Specific Quality of Life (THYCA-QoL) questionnaire (10). The THYCA-QoL questionnaire includes 24 questions measuring seven symptom domains and six individual scales. All responses are scored depending on four levels: 1 for “not at all”, 2 for “a little”, 3 for “quite a bit”, and 4 for “very much”. Given that ET via axillary approach may potentially damage the PMF and pectoralis major muscle and impair the motion of the shoulder joint, we additionally employed the American Shoulder and Elbow Surgeons Standardized Shoulder Assessment Form (ASES) questionnaire as a patient-reported outcome measure to quantitatively assess patients’ perceived pain levels and functional capacity following surgery (11, 12). All patients, including those in both Group A and Group B, completed the survey at 1 month, 3 months, and 6 months postoperatively.

### Statistical analysis

SPSS software version 25.0 (SPSS Inc., Chicago, IL) was used for statistical analyses. Categorical variables were presented as numbers, while continuous variables were expressed as mean ± standard deviation (SD). The Pearson’s chi-square test and Student’s *t*-test was employed to compare two categorical variables and continuous variables, respectively. Effect sizes were calculated as Cohen’s d using pooled standard deviations, and 95% confidence intervals were derived from the t distribution. A *post-hoc* power analysis was performed for the 6-month ASES total score using the observed effect size, group sample sizes, and α=0.05. The 6-month ASES total score was prespecified as the primary endpoint. Secondary endpoints included ASES subscales and THYCA-QoL domains at 1, 3, and 6 months. To control for multiple testing, we applied Bonferroni and Benjamini–Hochberg FDR corrections (q=0.05). All statistical tests were two-sided, and a *P* < 0.05 was considered statistically significant.

## Results

### Clinicopathological features and clinical outcomes

In this study, a total of 77 patients, 39 patients with PMF preservation in Group A and 38 patients without PMF preservation in Group B, were included for analysis. The median follow-up duration was 7.55 ± 1.36 (range, 6–10) months. The detailed clinicopathological features and clinical outcomes of the Group A and Group B are summarized in **Table 1**. The comparison results of the baseline characteristics showed no significantly different, except the total postoperative drainage of Group A was lower than that of Group B (A: 121.58 ± 33.22 vs. B: 168.76 ± 50.11, p <0.001). No between-group difference was observed in clinically significant postoperative hematoma events, likely due to the low baseline incidence.

**Table 1:** Baseline characteristics and perioperative outcomes of group A and group B.

Characteristics		Group A (n=39)	Group B (n=38)	*P*
Sex				
	Female	31	29	0.63
	Male	8	9	
Age (years)		40.05 ± 15.78	41.82 ± 15.99	0.62
	<55	31	30	0.96
	≥55	8	8	
Weight (kg)		58.41 ± 7.66	58.01 ± 11.32	0.85
BMI (kg/m2)		22.24 ± 3.60	23.10 ± 4.68	0.29
Family history				
	Yes	4	3	0.72
	No	35	35	
Operative duration (min)		95.77 ± 10.06	95.42 ± 16.45	0.88
Tumor size (cm)		0.95 0.38	0.92 0.33	0.71
Multifocality				
	Yes	5	5	0.97
	No	34	33	
Pathological type				
	Malignant	36	34	0.66
	Benign	3	4	
Temporary RLN paralysis				
	Yes	1	1	0.99
	No	38	37	
Total postoperative drainage (ml)		121.58 ± 33.22	168.76 ± 50.11	**<0.001**
Hospital stay (days)		5.47 ± 0.82	5.68 ± 1.16	0.38
Follow-up times (months)		7.57 ± 1.20	7.72 ± 1.54	0.52

Bold values were <0.05 considered statistically significance.

### THYCA-QoL and ASES questionnaires outcomes

The results of the comparative analysis between Group A and Group B in terms of THYCA-QoL questionnaire at 1 month, 3 months, and 6 months are presented in **Table 2**. At 1 month and 3 months postoperatively, no significant differences were observed in THYCA-QoL scores between groups. By 6 months, there were three differences in various aspects of the THYCA-QoL questionnaire, including neuromuscular (A: 1.51 ± 0.72 vs. B: 1.84 ± 0.72, p = 0.03), sympathetic (A: 1.38 ± 0.63 vs. B: 1.79 ± 0.78, p = 0.01), and sensory (A: 1.44 ± 0.72 vs. B: 1.87 ± 0.78, p = 0.01) between groups (**Table 2**).

**Table 2:** Comparison analysis of quality of life based on THYCA QoL questionnaire.

Scores	1 month		*P*	3 months		*P*	6 months		*P*
	Group A	Group B		Group A	Group B		Group A	Group B	
Neuromuscular	2.10 ± 0.97	2.08 ± 0.82	0.91	1.74 ± 0.85	2.00 ± 0.77	0.17	1.51 ± 0.72	1.84 ± 0.72	**0.03**
Voice	1.79 ± 0.89	1.74 ± 0.64	0.75	1.51 ± 0.64	1.61 ± 0.75	0.56	1.44 ± 0.64	1.42 ± 0.64	0.92
Concentration	1.64 ± 0.84	1.61 ± 0.64	0.83	1.67 ± 0.77	1.63 ± 0.79	0.84	1.41 ± 0.55	1.42 ± 0.68	0.94
Sympathetic	2.05 ± 0.89	2.13 ± 0.88	0.69	1.69 ± 0.89	1.95 ± 0.84	0.20	1.38 ± 0.63	1.79 ± 0.78	**0.01**
Throat/mouth	1.82 ± 0.76	1.82 ± 0.73	0.98	1.56 ± 0.79	1.61 ± 0.68	0.81	1.46 ± 0.55	1.45 ± 0.65	0.92
Psychological	1.69 ± 0.86	1.74 ± 0.86	0.82	1.59 ± 0.82	1.68 ± 0.70	0.59	1.56 ± 0.82	1.47 ± 0.65	0.59
Sensory	2.08 ± 0.96	1.97 ± 0.82	0.61	1.67 ± 0.81	1.92 ± 0.82	0.17	1.44 ± 0.72	1.87 ± 0.78	**0.01**
Problems with scar	1.56 ± 0.68	1.82 ± 0.87	0.16	1.59 ± 0.75	1.63 ± 0.67	0.80	1.59 ± 0.64	1.61 ± 0.68	0.92
Felt chilly	1.41 ± 0.72	1.39 ± 0.64	0.92	1.33 ± 0.53	1.37 ± 0.59	0.78	1.33 ± 0.62	1.34 ± 0.58	0.95
Tingling hands/feet	1.41 ± 0.67	1.55 ± 0.76	0.39	1.36 ± 0.58	1.58 ± 0.79	0.17	1.31 ± 0.47	1.29 ± 0.46	0.86
Gained weight	1.44 ± 0.68	1.50 ± 0.76	0.70	1.54 ± 0.76	1.55 ± 0.76	0.93	1.74 ± 0.82	1.76 ± 0.82	0.92
Headache	1.56 ± 0.64	1.61 ± 0.75	0.80	1.51 ± 0.68	1.47 ± 0.73	0.81	1.49 ± 0.72	1.45 ± 0.69	0.80
Interested in sex	1.97 ± 0.90	1.84 ± 0.89	0.52	2.08 ± 0.98	2.11 ± 0.92	0.90	2.15 ± 0.96	2.11 ± 0.76	0.81

*THYCA QOL, Thyroid Cancer-Specific Quality of Life.

Bold values were <0.05 considered statistically significance.

To evaluate the effects of preservation of PMF during the surgery on the shoulder joint, ASES questionnaire survey was conducted in all enrolled cases. As results, the ASES sores in terms of pain and function at 1 month and 3 months had no significantly different. However, at 3 months, Group A had higher total ASES score than Group B (A: 73.92 ± 10.81 vs. B: 67.39 ± 12.38, p = 0.02). By 6 months, Group A outperformed Group B in total score (A: 94.62 ± 6.81 vs. B: 86.74 ± 9.62, p < 0.001), pain (A: 47.05 ± 5.59 vs. B: 44.08 ± 6.96, p = 0.04), and function (A: 47.57 ± 3.43 vs. 42.66 ± 6.25, p < 0.001), underscoring the benefits of PMF preservation on shoulder stability and mobility (**Table 3**). At 6 months, the mean ASES total score was 90.3 ± 20.7 in the preservation group (n = 42) and 84.8 ± 15.4 in the non-preservation group (n = 40), with a mean difference of 5.46 points (95% CI = 2.47 to 13.39). The standardized effect size was small to moderate (Cohen’s d = 0.30), corresponding to a *post-hoc* statistical power of approximately 0.40 at α=0.05. While some QoL domains and ASES subscales showed nominal significance before correction, only the 6-month ASES total score and the 6-month ASES function subscale remained significant after Bonferroni and FDR adjustments. Other QoL differences lost significance after correction.

**Table 3:** Comparison analysis of the effects of treatment on shoulder joint based on ASES.

Scores	1 month		*P*	3 months		*P*	6 months		*P*
	Group A	Group B		Group A	Group B		Group A	Group B	
Total	53.33 ± 11.20	52.36 ± 10.78	0.70	73.92 ± 10.81	67.39 ± 12.38	**0.02**	94.62 ± 6.81	86.74 ± 9.62	**<0.001**
Pain	31.67 ± 9.34	31.45 ± 7.44	0.91	36.54 ± 9.12	33.03 ± 10.04	0.11	47.05 ± 5.59	44.08 ± 6.96	**0.04**
Function	21.66 ± 8.48	20.91 ± 7.92	0.69	37.38 ± 6.58	34.37 ± 7.23	0.06	47.57 ± 3.43	42.66 ± 6.25	**<0.001**

*ASES, American Shoulder and Elbow Surgeons Standardized Shoulder Assessment Form.

Bold values were <0.05 considered statistically significance.

## Discussion

ET via axillary approach provides superior cosmesis and reduces neck-related morbidity compared to conventional open surgery, especially for patients with high aesthetic demands (2, 13). Nonetheless, this approach necessitates meticulous dissection through the PMF to access the thyroid bed, which may inadvertently damage sensory nerves (e.g., supraclavicular nerve branches) and musculoskeletal structures, leading to postoperative sensory deficits and functional impairment (14). However, this technique requires meticulous dissection through the PMF, which harbors critical neurovascular structures. In our study, preservation of the PMF was associated with significantly better shoulder function at 6 months, as reflected in both ASES total and function scores. Although we did not directly measure sympathetic or neuromuscular recovery, there is anatomical and physiological plausibility supporting this association. The PMF provides a protective layer for vascular and neural structures and serves as a gliding plane between the skin flap and underlying muscle. Disruption of this fascia may increase mechanical stress, promote adhesion, and expose supraclavicular nerve branches that innervate the anterior chest and shoulder, thereby contributing to neurosensory disturbances. Anatomical studies have described the variability and vulnerability of these nerve branches during cervical and chest wall dissection (15). Moreover, surgical reports on gasless transaxillary endoscopic thyroidectomy emphasize the importance of maintaining fascial integrity to minimize postoperative discomfort and sensory impairment (5, 9). Taken together, these observations provide a biologically plausible framework for why PMF preservation may favor improved neurosensory outcomes. Nevertheless, we acknowledge that these mechanisms remain hypothetical, and further experimental or neurophysiological studies are warranted to confirm this link.

Beyond neurosensory recovery, our findings also showed that PMF preservation was associated with reduced postoperative drainage. The PMF represents a critical anatomical boundary, providing structural support to the pectoralis major muscle while encasing sensory nerve fibers and perforating branches of the thoracoacromial artery, which are susceptible to traction or cautery injury. Disruption of this layer may increase exudation and bleeding. Meticulous hemostasis and use of ultrasonic devices may help mitigate such risks. However, attributing increased drainage solely to PMF disruption may be an oversimplification. Other perioperative factors, such as subtle variations in hemostasis technique, differences in patient comorbidities (e.g., hypertension), coagulation status, or slight extension of dissection margins, may also contribute. Previous studies of thyroid surgery have identified advanced age, male sex, higher BMI, extent of neck dissection, drain placement, and longer operative time as independent risk factors for postoperative hematoma or bleeding (16–18). In our study, groups were balanced in baseline characteristics and all procedures were performed by the same team, but residual confounding by these factors cannot be excluded. Thus, PMF preservation should be regarded as one plausible contributor to reduced drainage rather than the sole determinant.

The THYCA-QoL results revealed persistent neuromuscular, sympathetic and sensory symptoms in Group B at the 6-month follow-up. This finding aligns with Zhou et al.’s observation that supraclavicular nerve (SCN) injury during ET via anterior chest approach leads to prolonged anterior chest hypoesthesia (19). The PMF serves as a conduit for medial SCN branches (15) and their disruption during axillary dissection potentially accounted for the sensory deficits (20, 21). The differences in the term of symptoms of neuromuscular, sympathetic, and sensory might due to the variations in sensory nerve regeneration (e.g., A-δ fibers), which often requires 6 to 12 months, explaining delayed improvements observed in Group A.

The inferior ASES scores in Group B suggest PMF disruption compromises the pectoralis major’s biomechanical function, exacerbating postoperative shoulder pain and limiting mobility. Song et al. similarly reported that reduced shoulder morbidity following axillary approach thyroidectomy, which was probably caused by the PMF retraction injury (22). The PMF anchors the clavicular head of the pectoralis major, while its violation may alter muscle kinematics, leading to the regional pain and functional impairment (23). Early ASES scores declined in the both group were related to the acute tissue injury and inflammation resulting from the axillary approach traversing the PMF. However, for patients with PMF preservation in Group A, their shoulder function gradually recovered by 3 months whereas those in Group B experienced delayed recovery of surgical injuries, resulting in significantly differences at the 6-month follow-up.

This study has some limitations that should be acknowledged. First, its retrospective nature with limited cases and non-randomized patient allocation may introduce potential bias. Although a trend toward improved shoulder function was observed in the preservation group, the effect size was modest and the *post-hoc* power analysis indicated that our study had only about 40% power to detect this difference. Thus, the present findings should be interpreted with caution, and larger prospective studies are needed to confirm the potential clinical benefit of PMF preservation. Another limitation of our study is the relatively short follow-up duration of 6 months. This time frame may be insufficient to fully capture long-term neurosensory or functional recovery. Reports of robotic and transaxillary thyroidectomy have indicated that anterior chest wall sensory disturbance and discomfort may persist for 12 months or longer before gradual improvement (24). Accordingly, our results should be regarded as mid-term outcomes, and longer follow-up is necessary to validate the durability of PMF preservation benefits. Because of the modest sample size, propensity score–based methods (PSM/IPTW) were not feasible, which may leave residual confounding. Future larger multicenter cohorts will allow more advanced bias-reduction techniques to assess the clinical benefits of PMF preservation of these patients.

## Conclusion

Our findings demonstrate that PMF preservation significantly improves QoL, reduces postoperative bleeding, as well as enhances long-term recovery of sensory and shoulder function for patients undergoing ET via the axillary approach. In light of these findings, surgeons may make every effort to maintain the integrity of PMF during the surgical procedure.

## Data Availability

The raw data supporting the conclusions of this article will be made available by the authors, without undue reservation.
